# Predictive Role of Serum Cytokine Profiles in Acute Kidney Injury after Living Donor Liver Transplantation

**DOI:** 10.1155/2018/8256193

**Published:** 2018-04-01

**Authors:** Min Suk Chae, Youngchan Kim, Hyun Sik Chung, Chul Soo Park, Jaemin Lee, Jong Ho Choi, Sang Hyun Hong

**Affiliations:** Department of Anesthesiology and Pain Medicine, Seoul St. Mary's Hospital, College of Medicine, The Catholic University of Korea, Seoul, Republic of Korea

## Abstract

**Introduction:**

Previous studies have shown that a higher serum interleukin- (IL-) 6 level is associated with a higher risk of acute kidney injury (AKI) development after major nontransplant surgery. Our study investigated the potential association of preoperative serum cytokine profiles with new AKI development in patients who underwent living donor liver transplantation (LDLT).

**Methods:**

Serum levels of cytokines IL-2, IL-6, IL-10, IL-12, and IL-17, interferon-*γ*, and tumor necrosis factor- (TNF-) *α* were measured in 226 LDLT recipients preoperatively and analyzed retrospectively. Recipients with a preoperative functional impairment of the kidney were excluded. AKI was defined according to Kidney Disease: Improving Global Outcomes (KDIGO) criteria.

**Results:**

In a univariate regression model, IL-6, IL-17, and TNF-*α* levels showed an association with AKI development after LDLT. Multivariate analysis showed an independent association of the preoperative serum IL-6 level with AKI development after LDLT and a significant relationship between higher serum IL-6 levels and a greater likelihood of developing AKI. Serum IL-6 levels were higher in patients with stage 3 AKI than in patients who did not develop AKI.

**Conclusions:**

Our results support the need for further investigations of IL-6 as a predictor of AKI development in patients undergoing LDLT.

## 1. Introduction

Acute kidney injury (AKI) is one of the most common complications after liver transplantation. Although the risk of developing postoperative AKI is lower in living donor liver transplantation (LDLT) patients than in patients transplanted with a liver from a deceased donor, in both groups, it is still a major cause of an adverse outcome [1]. Among the clinical factors that influence AKI development in LDLT patients are immunosuppressant use, blood loss, blood product transfusion during surgery, and hemodynamic instability [2, 3].

Cytokines are multifunctional proteins with important roles as intracellular molecules. They are released by immune cells, including T-cells and macrophages, and include interleukins IL-1, IL-2, IL-6, IL-12, and IL-17, interferon- (IFN-) *γ*, and tumor necrosis factor- (TNF-) *α*, all of which promote inflammation, and IL-4, IL-10, IL-11, and IL-13, which exhibit anti-inflammatory activity [4, 5]. Severe inflammation is the product of an imbalance between pro- and anti-inflammatory cytokines and underlies the development of liver cirrhosis and fibrosis, leading to end-stage liver disease (ESLD) [6]. Kidney cells, such as podocytes, mesangial cells, endothelial cells, and tubular epithelial cells, also secrete cytokines that, via immunologic responses, play an important role in inducing the injury or repair of the diseased kidney, whether mediated by immunologic, ischemic, metabolic, or toxigenic factors [7–9].

Little is known about the predictive role of serum cytokine profiles with respect to AKI development after LDLT. Thus, in the present study, we investigated the association of preoperative serum cytokine profiles with both new AKI development after LDLT and the severity of postoperative AKI.

## 2. Patients and Methods

### 2.1. Study Population

The study population consisted of 244 adult patients (age ≥ 19 years) with ESLD who underwent LDLT from September 2010 to July 2014 at Seoul St. Mary's Hospital (Republic of Korea). The clinical exclusion criteria were functional impairment of the kidney before surgery, such as acute or chronic kidney injury, hepatorenal syndrome, or a history of hemodialysis. The perioperative data of the recipients and donors were reviewed retrospectively using the hospital electronic medical records system. The Institutional Review Board of the Seoul St. Mary's Hospital Ethics Committee approved this study (KC17RISI0001). The need for informed consent was waived.

### 2.2. Perioperative Patient Management

LDLT was performed using the right hepatic lobe and the piggyback technique, including middle hepatic vein reconstruction, in patients administered balanced anesthesia, as described previously [10]. The hemodynamic status of the patients was maintained using appropriate fluid resuscitation and inotropic administration under invasive vital sign monitoring.

During the perioperative period, the patients were kept on an immunosuppression regimen, including tacrolimus, mycophenolate mofetil (MMF), prednisolone, and basiliximab, based on the LDLT protocol of our hospital. The trough level of tacrolimus was maintained between 7 and 10 ng/mL for the first month after LDLT and between 5 and 7 ng/mL thereafter. The trough level of cyclosporine was maintained between 100 and 150 ng/mL for the first month after LDLT and between 50 and 100 ng/mL thereafter. Steroids were gradually tapered within the first month after LDLT, while MMF was tapered between 3 and 6 months after LDLT. An IL-2 receptor blocker, such as basiliximab, was infused on the day before LDLT surgery and on day 4 after surgery [11].

### 2.3. Definition of Acute Kidney Injury

In this study, AKI was defined according to the Kidney Disease: Improving Global Outcomes (KDIGO) criteria [12]: increase in serum creatinine (sCr) ≥ 0.3 mg/dL (≥26.5 *μ*mol/L) by POD 2 or an increase in sCr ≥ 1.5 times the baseline within the first week after surgery.

The severity of AKI was staged as follows: stage 1 disease was a sCr level 1.5–1.9 times the baseline or an increase of ≥0.3 mg/dL (≥26.5 *μ*mol/L); stage 2 was a sCr level 2.0–2.9 times the baseline; and stage 3 was a SCr level 3.0 times the baseline or an increase of ≥4.0 mg/dL (≥353.6 *μ*mol/L) or renal replacement therapy.

According to AKI development, patients were assigned to either the non-AKI or the AKI group.

### 2.4. Measurement of Serum Cytokines

The serum profiles of the cytokines IL-2, IL-6, IL-10, IL-12, and 17, IFN-*γ*, and TNF-*α* were determined in all transplant recipients who underwent elective LDLT on the day before surgery. The cytokine immunoassays were carried out using blood samples collected under sterile conditions in BD Vacutainer tubes containing K2EDTA (Becton, Dickinson, Franklin Lakes, NJ). After their transfer to the laboratory in an ice-filled container, the samples were centrifuged (1500 rpm, 10 min, 4°C), frozen at −70°C, and stored until analyzed using a sandwich enzyme-linked immunosorbent assay with a human 25-plex antibody bead kit (Invitrogen, Camarillo, CA). The data were analyzed using a Luminex 200 detection system (Luminex, Austin, TX).

### 2.5. Perioperative Data

Preoperative recipient factors included age, sex, body mass index (BMI), etiology of the need for LDLT, model for end-stage liver disease (MELD) score, hepatic decompensation complications, and laboratory variables. Intraoperative recipient factors included total operation time; incidence of severe postreperfusion syndrome, assessed based on the occurrence of severe hemodynamic instability, fatal arrhythmia, requirement for strong vasopressors, and prolonged or recurrent fibrinolysis [13]; blood product transfusions, hourly fluid infusions and urine output; and dose of furosemide administered. Donor-graft factors included age, sex, BMI, graft volume to standard liver volume ratio at the time of transplantation, graft fat percentage, and total ischemic time.

### 2.6. Statistical Analysis

Continuous data are presented as the median (interquartile range (IQR)) and were compared using the Mann–Whitney *U* test. Categorical data are expressed as a number (proportion) and were assessed using a *χ*^2^ test or Fisher's exact test, as appropriate. The Kruskal–Wallis test with a Bonferroni post hoc test was used to compare preoperative serum cytokine levels with the severity of AKI. The association between the preoperative serum cytokine profile, dichotomized into high and low according to the median values, and AKI development was analyzed in a univariate logistic regression. Potentially significant factors (*p* < 0.1) were assessed in a predictive model using multivariate logistic regression. All tests were two-sided and a *p* value < 0.05 was considered to indicate statistical significance. The statistical analyses were performed using SPSS (ver. 24.0 for Windows; SPSS Inc., Chicago, IL, USA) and MEDCALC (ver. 11.0 for Windows; MedCalc Software, Mariakerke, Belgium).

## 3. Results

Eighteen patients were excluded based on the exclusion criteria, including acute or chronic kidney injury (*n* = 6), hepatorenal syndrome (*n* = 4), and a history of hemodialysis (*n* = 4). Serum cytokine profile data were missing for additional four patients. Therefore, the data of 226 patients were investigated. Within this group, 173 (76.5%) patients had normal kidney function after LDLT and 53 (23.5%) patients developed AKI postoperatively. In the latter, the severity was as follows: 33 (14.6%) patients had stage 1, 12 (5.3%) had stage 2, and 8 (3.5%) had stage 3 disease. The study cohort was predominantly (70.4%) male; the median (IQR) age was 53 (48–59) years, and the median (IQR) BMI was 24.2 (22.0–26.4) kg/m^2^. The most common indication for LDLT was viral hepatitis, including hepatitis B (61.1%) and C (5.3%), followed by alcohol abuse (19.5%), drug- or toxin-related hepatitis (6.6%), autoimmune hepatitis (2.2%), and cryptogenic hepatitis (5.3%). The median (IQR) MELD score was 15 (9–23) points. Hepatic decompensation complications included encephalopathy (6.2%), varix (25.2%), and ascites (41.2%).

Among the preoperative findings, male recipients constituted a larger proportion of the AKI than the non-AKI group (Table 1). The sCr level was higher, and the estimated glomerular filtration rate (eGFR) was lower, in the AKI group compared to the non-AKI group; however, the sCr and eGFR levels were within the normal range in both groups [14]. C-reactive protein (CRP) levels were higher, and platelet counts were lower, in the AKI group than in the non-AKI group. Intraoperatively, the incidence of severe postreperfusion syndrome was higher and blood products, including fresh frozen plasma and platelet concentrate, were more often transfused in AKI than in non-AKI patients. Liver donors were older in the AKI group than in the non-AKI group.

Table 2 shows the cytokine levels of AKI and non-AKI patients on the day prior to LDLT. Serum IL-6 and TNF-*α* levels on the preoperative day were significantly higher in the AKI group versus the levels in the non-AKI group after LDLT.

**Figure 1:**
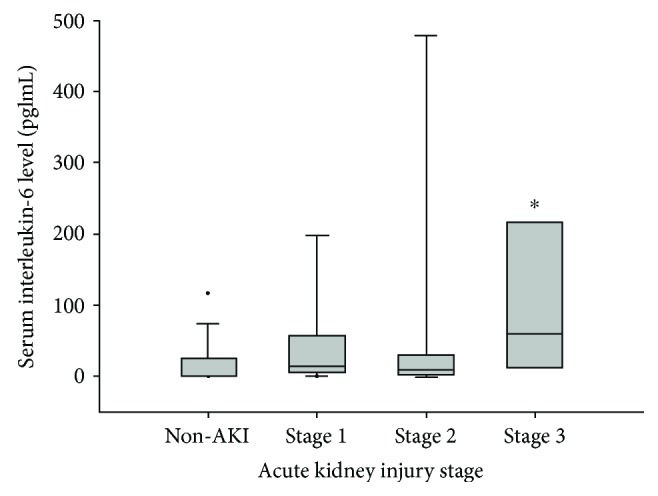
Comparison of the preoperative serum interleukin-6 levels among patients with postoperative acute kidney injury staged according to the Kidney Disease: Improving Global Outcomes (KDIGO) criteria. The box plots show the median (line in the middle of the box), interquartile range (box), 5th and 95th percentiles (whiskers), and outliers (dots). ^∗^*p* < 0.05 versus no AKI development.

Dichotomization of the serum cytokine profiles into above versus below the median showed that, in the initial univariate regression model, IL-6, IL-17, and TNF-*α* were potentially associated with AKI development after LDLT (Table 3). In the multivariate regression analysis, the dichotomized serum IL-6 level was significantly associated with postoperative AKI development. An analysis of the AKI stage and serum IL-6 levels (Figure 1) showed significantly higher IL-6 levels in stage 3 AKI patients than in non-AKI patients.

## 4. Discussion

The main finding of this study was the independent association of the preoperative serum IL-6 level with AKI development after LDLT. Higher serum IL-6 levels were significantly related to a greater likelihood of developing AKI and were more often detected in patients with stage 3 AKI than in non-AKI patients.

IL-6 is produced immediately in wounded tissues during the early stage of the inflammatory response. It promotes the differentiation and activation of T-cells, B-cells, and macrophages in response to infection and tissue damage [15]. The pleiotropic effect of IL-6 in the inflammatory immune response includes stimulation of the production of acute-phase proteins, including CRP, fibrinogen, and serum amyloid A, and suppression of the synthesis of albumin, fibronectin, and transferrin [16]. In liver homeostasis, IL-6 plays an important role in protecting hepatocytes against ischemia-reperfusion injury and induces liver regeneration and repair [17, 18]. In liver transplant patients, a higher preoperative serum IL-6 level is associated with a lower risk of the postoperative development of early allograft dysfunction [19]. However, the dysregulated production of IL-6 is closely related to pathologic outcomes in patients with chronic inflammatory and autoimmune conditions [20]. In patients with ESLD, high blood levels of IL-6 are associated with worse survival. Moreover, the predictive value of the serum IL-6 level for patient mortality was shown to be similar to that of the MELD score but it was higher than that of other inflammatory markers, including CRP and the white blood cell count [21].

Further evidence for a link between IL-6 and kidney disorder is the inverse association of the eGFR with the circulating levels of proinflammatory markers, including IL-6. The eGFR was significantly lower in patients with levels of circulating inflammatory markers, such as IL-6, that were higher than those of the general population [22]. In pediatric cardiac surgery, the preoperative serum IL-6 level is associated with postoperative AKI development and patients with IL-6 concentrations in the upper tertile have a six times higher risk of developing AKI stages 2 and 3 than do patients with IL-6 levels in the lower tertile [23]. In adult cardiac surgery patients, increased plasma IL-6 levels on POD 1 are associated with AKI development, and the risk of AKI increases with increasing IL-6 levels [24]. These studies highlight the crucial role of inflammation in the pathophysiology of AKI. In our study, a higher preoperative serum IL-6 was independently related to a greater likelihood of AKI development, although why patients scheduled for LDLT differ in their preoperative serum IL-6 levels is not yet known. As in previous studies [23, 24], our result showed that patients with postoperative AKI stage 3 had significantly higher preoperative serum IL-6 levels than those without AKI. Taken together, these findings suggest that the preoperative serum IL-6, as a major biomarker of inflammation, can serve as a clinically relevant predictor of AKI development in patients undergoing LDLT.

Our study also had several limitations. Firstly, the exact source of cytokine synthesis giving rise to the high serum levels could not be identified, because patients with ESLD suffer from multiple organ injuries. However, because cytokines within damaged tissues are released into the systemic circulation, their levels can presumably be determined by measuring circulating serum cytokines in the peripheral blood and, in this study, they provided an estimate of the degree of inflammation in LDLT patients. Secondly, the effect of immunosuppressants on serum cytokine synthesis could not be measured directly due to the retrospective design of the study. All patients who underwent LDLT were placed on an immunosuppression regimen, in accordance with the LDLT protocol of our hospital, which might have affected cytokine expression. Finally, a cutoff value for the serum IL-6 level that predicted AKI development after LDLT was not calculated but should be determined in further studies, together with a reference range.

In conclusion, the present study identified IL-6 as a promising marker for predicting AKI development in LDLT patients. The reason for the preoperative difference in the serum IL-6 level between patients with and without AKI in the early postoperative period is not yet known. Serum IL-6 plays important roles in liver homeostasis, for example, with respect to the proliferation and repair of hepatocytes after injury. Balancing the perioperative IL-6 level to promote hepatocyte proliferation while preventing AKI development after LDLT is a goal for the future.

## Figures and Tables

**Table 1:** Comparison of perioperative findings between patients with and without acute kidney injury after living donor liver transplantation.

*n*	Non-AKI group	AKI group	*p*
	173	53	
*Preoperative recipient factor*			
Age (years)	53 (48–59)	53 (49–60)	0.728
Gender (male)	115 (66.5%)	44 (83.0%)	0.021
Body mass index (kg/m^2^)	24.1 (21.8–25.9)	24.4 (22.2–28.1)	0.222
*Etiology*			0.173
Alcohol	32 (18.5%)	12 (22.6%)	
Hepatitis B	102 (59.0%)	36 (67.9%)	
Hepatitis C	11 (6.4%)	1 (1.9%)	
Autoimmune	5 (2.9%)	0 (0.0%)	
Drug & toxin	11 (6.4%)	4 (7.5%)	
Cryptogenic findings	12 (6.9%)	0 (0.0%)	
MELD score (points)	14 (9–22)	15 (10–25)	0.275
*Complications of hepatic decompression*			
Severe encephalopathy^∗^	12 (6.9%)	2 (3.8%)	0.528
Varix	44 (25.4%)	13 (24.5%)	0.878
Ascites (≥1 L)	67 (38.7%)	26 (49.1%)	0.181
*Laboratory value*			
Hematocrit (%)	30.1 (25.2–36.3)	28.4 (25.3–32.2)	0.146
Sodium (mEq/L)	139.0 (135.0–142.0)	139.0 (136.0–141.0)	0.763
Creatinine (mg/dL)	0.8 (0.7–1.0)	0.7 (0.6–0.9)	0.015
MDRD GFR (mL/min/1.73 m^2^)	96 (72–128)	117 (86–158)	0.015
C-reactive protein (mg/dL)	0.3 (0.1–0.9)	0.7 (0.2–1.7)	0.004
Platelet (×10^9^/L)	67.0 (47.0–119.5)	52.0 (38.5–77.0)	0.006
Total bilirubin (mg/dL)	2.0 (0.8–6.7)	2.5 (1.1–8.3)	0.354
International normalized ratio	1.4 (1.2–1.8)	1.6 (1.3–1.9)	0.093
*Intraoperative recipient factor*			
Total operation duration (min)	525 (455–574)	495 (465–560)	0.522
Severe postreperfusion syndrome	28 (16.2%)	19 (35.8%)	0.002
*Blood product transfusion (unit)*			
Packed red blood cell	6 (3–11)	8 (5–12)	0.057
Fresh frozen plasma	6 (4–10)	8 (5–13)	0.006
Platelet concentrate	0 (0–6)	5 (0–10)	0.035
Cryoprecipitate	0 (0–0)	0 (0–0)	0.090
Hourly fluid administration (mL/kg/h)	10.1 (7.9–12.6)	8.2 (6.4–13.1)	0.174
Hourly urine output (mL/kg/h)	1.4 (0.8–2.2)	1.1 (0.7–1.9)	0.085
Furosemide administration (mg)	5 (0–20)	10 (0–33)	0.094
*Donor-graft factor*			
Age (years)	31 (24–41)	36 (28–48)	0.027
Gender (male)	76 (43.9%)	21 (39.6%)	0.524
Body mass index (kg/m^2^)	23.0 (21.1–25.4)	23.0 (21.4–25.9)	0.587
Graft volume to standard liver volume ratio at time of transplantation (%)	55.4 (48.5–65.4)	53.5 (47.3–64.4)	0.505
Steatosis percentage (%)	3.0 (0.0–5.0)	4.0 (0.0–5.0)	0.958
Total ischemic time (min)	98 (71–124)	102 (70–158)	0.366

AKI: acute kidney injury; MELD: model for end-stage liver disease; MDRD: the Modification of Diet in Renal Disease Study equation; GFR: glomerular filtration rate. ^∗^Severe encephalopathy: West-Haven criteria III or IV. Values are expressed as numbers (portions) and median (interquartile).

**Table 2:** Comparison of serum cytokine levels on preoperative day between patients with and without acute kidney injury after living donor liver transplantation.

*n*	Whole study group	Non-AKI group	AKI group	*p*
	226	173	53	
*Serum cytokine levels (pg/mL)*				
Interleukin-2	0.1 (0.1–1.6)	0.1 (0.1–1.6)	0.1 (0.1–1.4)	0.956
Interleukin-6	7.3 (0.1–30.2)	5.8 (0.1–24.5)	12.7 (3.7–53.1)	0.018
Interleukin-10	0.5 (0.1–11.0)	0.4 (0.1–7.5)	3.5 (0.1–30.8)	0.068
Interleukin-12	0.1 (0.1–0.1)	0.1 (0.1–0.1)	0.1 (0.1–0.1)	0.630
Interleukin-17	2.0 (0.1–16.4)	1.5 (0.1–12.7)	4.3 (0.1–20.2)	0.147
Interferon-*γ*	3.2 (0.1–16.0)	3.2 (0.1–19.2)	3.2 (0.1–10.3)	0.764
Tumor necrosis factor-*α*	9.8 (5.4–17.9)	9.3 (5.1–16.6)	13.3 (6.2–23.6)	0.040

Values are expressed as numbers (portions) and median (interquartile).

**Table 3:** Association between preoperative serum cytokine profiles and postoperative acute kidney injury development after living donor liver transplantation using univariate and multivariate logistic regression.

		Univariate logistic analysis				Multivariate logistic analysis			
Serum cytokine level (pg/mL)	Median	*β*	Odds ratio	95% CI	*p*	*β*	Odds ratio	95% CI	*p*
Interleukin-2	0.1	0.161	1.175	(0.624–2.211)	0.617				
Interleukin-6	7.3	0.862	2.368	(1.246–4.503)	0.009	0.862	2.368	(1.246–4.503)	0.009
Interleukin-10	0.5	0.37	1.448	(0.779–2.690)	0.242				
Interleukin-12	0.1	−0.008	0.992	(0.400–2.461)	0.987				
Interleukin-17	2.0	0.572	1.771	(0.947–3.314)	0.074				
Interferon-*γ*	3.2	0.043	1.044	(0.564–1.933)	0.891				
Tumor necrosis factor-*α*	9.8	0.572	1.771	(0.947–3.314)	0.074				

The serum cytokine profiles were dichotomized at the median into high and low levels.

## Data Availability

The data used to support the findings of this study are available from the corresponding author upon request.
